# Flood disturbance affects morphology and reproduction of woody riparian plants

**DOI:** 10.1038/s41598-021-95543-0

**Published:** 2021-08-13

**Authors:** Sarah Fischer, Joe Greet, Christopher J. Walsh, Jane A. Catford

**Affiliations:** 1 School of Ecosystem and Forest Sciences, Burnley Campus, The University of Melbourne, 500 Yarra Bld, Richmond, 3121 Victoria Australia; 2 Department of Geography, King’s College London, 40 Aldwych, London, WC2B 4BG UK; 3 School of BioSciences, The University of Melbourne, Royal Parade, Parkville, 3052 Victoria Australia

**Keywords:** Forest ecology, Riparian ecology, Wetlands ecology, Ecology, Ecology

## Abstract

Riparian forests are structured and maintained by their hydrology. Woody riparian plants typically adapt to the local flood regime to maximise their likelihood of survival and reproductive success. Understanding how extant trees form and reproduce in response to flood disturbance is crucial for predicting vegetation changes and informing restoration. Working in a temperate evergreen riparian forest, we aimed to determine whether disturbance-based responses of plants found in other ecosystems also typify woody plants in riparian forests where disturbances are often mild or chronic, non-lethal, annual events. Using plant surveys and 20-year modelled hydrological data, we examined whether (1) the morphology (main stem diameter, height, crown width, crown extent, stem leaning) and (2) reproduction type (sexual and asexual reproduction) and extent of three dominant woody species (*Eucalyptus camphora*, *Leptospermum lanigerum* and *Melaleuca squarrosa*) vary with flood regime (flood frequency and flood duration); and (3) whether different morphology is associated with different reproductive strategies. Increased flooding generally resulted in increased stem numbers and greater stem leaning—morphologies associated with asexual reproduction—of our study species. More frequent flooding also reduced plant size and sexual reproduction in *E. camphora*. Sexual reproduction in the studied species was more common in taller plants with single, more upright stems in good condition. Flexible morphology and plastic reproductive strategy may constitute an adaptation of trees to mild or chronic disturbance in floodplains. Our findings suggest that flood regime (i.e. variable frequency and duration of flooding events) is critical to the structural integrity and self-maintenance of species-diverse riparian forests.

## Introduction

Healthy riparian forests control sediment runoff, purify water, stabilize banks, regulate stream temperatures and are home to considerable biodiversity. As they are large and abundant, the physical form of trees and shrubs is pivotal to the overall structure and function of forests. A remarkable feature of woody plants is their flexible morphology in response to their environment^1^. In forested floodplains, hydrology poses a major shaping force on tree and shrub development. Once established, woody plants adjust energy and resource allocations to survival, growth and reproduction in response to the flood regime, increasing their longevity and likelihood of reproductive success^1,2^. With increasing human impacts globally, anthropogenic flow regulation has disrupted the natural water fluctuations under which riparian forests have evolved, leading to their decline^3^. The resulting negative consequences for the environment and society have motivated efforts to actively restore riparian ecosystems. However, inducing such recovery requires sound understanding of woody plant responses to flood regime.

Challenges in ascertaining hydro-biological relationships arise from the diverse responses of plants to flooding^4^. On the one hand, regular flooding can provide plentiful water and nutrients, which usually supports tree growth^5^. On the other hand, flooding may cause physical damage resulting from flowing water and smothering by sediments, potentially resulting in scars or stem breakage^6^. Furthermore, prolonged periods of shallow flooding or waterlogging may cause plants physiological stress arising from soil anoxia and toxicity, even forcing plants into leaning positions due to substrate bogginess^7,8^. Although riparian plants can often withstand such unfavourable conditions (if their frequency does not compromise recovery capacity), metabolic costs for survival and recovery typically enforces limited growth and size^9^. Branch dieback and regrowth shapes the crown architecture and overall growth shape of trees^10^. Responses may differ among co-occurring woody plants depending on species-specific adaptations, growth form and phenological cycles^11,12^.

Previous research on vegetation responses to flooding have typically focused on survival, adaptive traits, growth (e.g. of stem diameter or biomass), structural mechanics, dendrogeomorphology or vegetation community composition^6,13–16^. Whole-tree morphology has received comparatively little attention outside forestry, despite its significance for ecosystem structure and function^17^, and there is a particular lack of empirical evidence from evergreen temperate riparian species subjected to regular, slow and shallow flooding. Trees embody their individual life history in their form. Understanding how extant trees have formed and regenerate in response to past flood regimes may provide valuable information for restoring adequate conditions (i.e. flood regimes) to promote regeneration and the development of desired forest structures.

**Figure 1:**
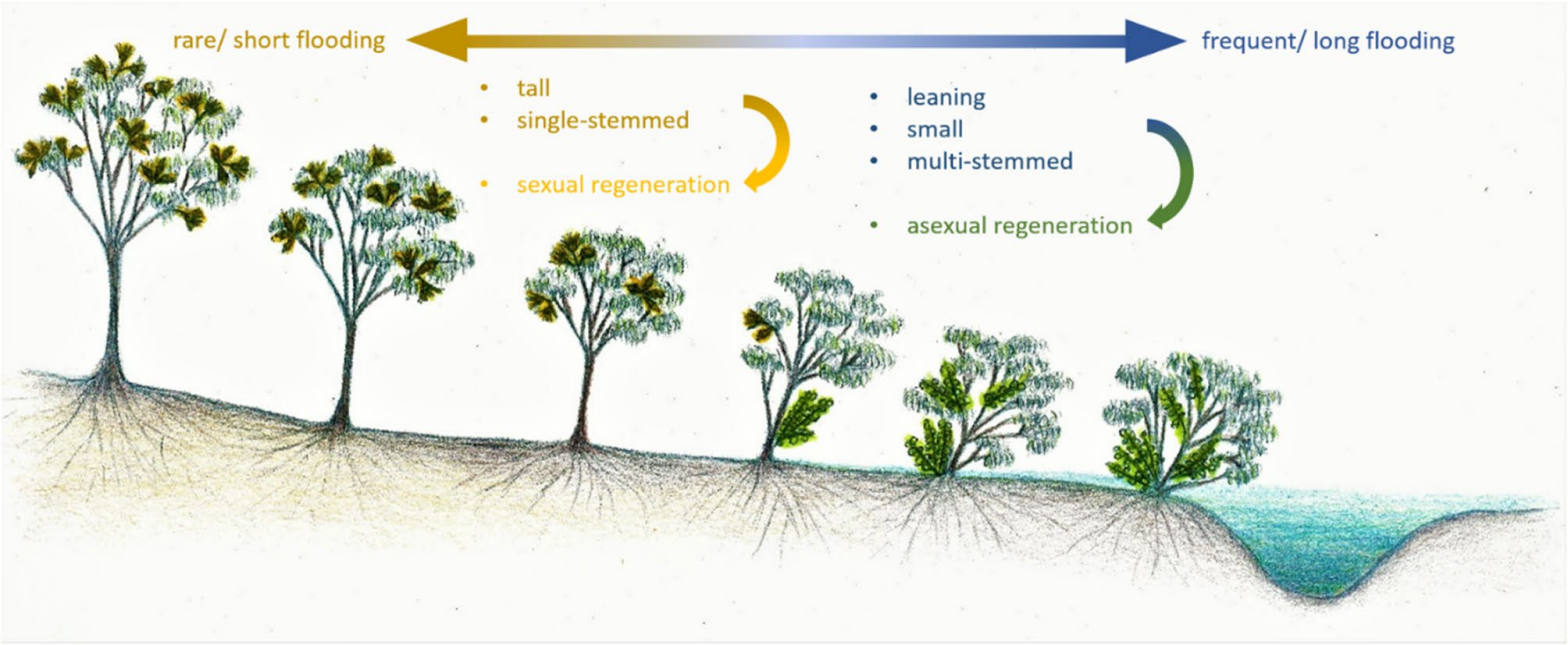
Hypothetical gradient in morphology and reproductive strategy of woody riparian plants in response to flood disturbance. Individuals that grow further from a channel or in higher elevations (left hand side) will experience less disturbance, i.e. shorter duration or less frequent flooding, which should hypothetically result in plants that are taller, single-stemmed and largely exhibit sexual or seed-based reproduction (symbolised by presence of flowers). In contrast, individuals that experience more disturbance (right hand side) will be more likely to have leaning stems, be shorter, multi-stemmed and reliant on vegetative reproduction (symbolised by epicormic resprouts).

Morphologic plasticity of perennial species in response to environmental factors has been well documented in other disturbance-prone ecosystems: mainly in those disturbed by fire^18^, but also in areas affected by catastrophic winds, landslides, avalanches^19–21^ and ephemeral and saline wetland systems^4,10,22^. Different morphologies are often associated with different reproductive strategies which are in turn influenced by the nature of the disturbance regime^23^. Two alternative strategies used by plants have been identified to maintain populations in disturbance-prone environments, namely resprouting and reseeding^24^, often also referred to as vegetative or asexual, and sexual reproduction^25^. Multi-stemmed, mostly smaller individuals often occur at sites with more disturbance and originate from and pursue vegetative persistence, whereas sexual or seed-based reproduction is more frequent in taller, usually single-stemmed individuals which experience less disturbance (Fig. 1). To date, the relationship between disturbance, morphology and reproductive strategy for woody plants in riparian forests remains unclear.

In this study, we aim to determine whether the disturbance-based responses of plants found in other ecosystems also typify woody plants in riparian forests where disturbances are moderate (i.e. low-energy floods which do not destroy or damage the majority of a plant’s biomass in one event) and occur regularly (e.g. seasonally each year). We surveyed morphological characteristics and extent of sexual and asexual reproduction in three woody riparian species in an evergreen temperate forest and examined their association with modelled, long-term flooding data to test the following hypothesis (Fig. 1):

(H1) : Morphology (main stem diameter, height, crown width, crown extent, stem leaning) varies with flood regime (flood frequency and flood duration);
(H2) : Reproduction type (sexual and asexual reproduction) and extent are plastic in response to flood regime;
(H3) : Different morphology is associated with different reproductive strategies.

## Methods

### Study site

**Figure 2:**
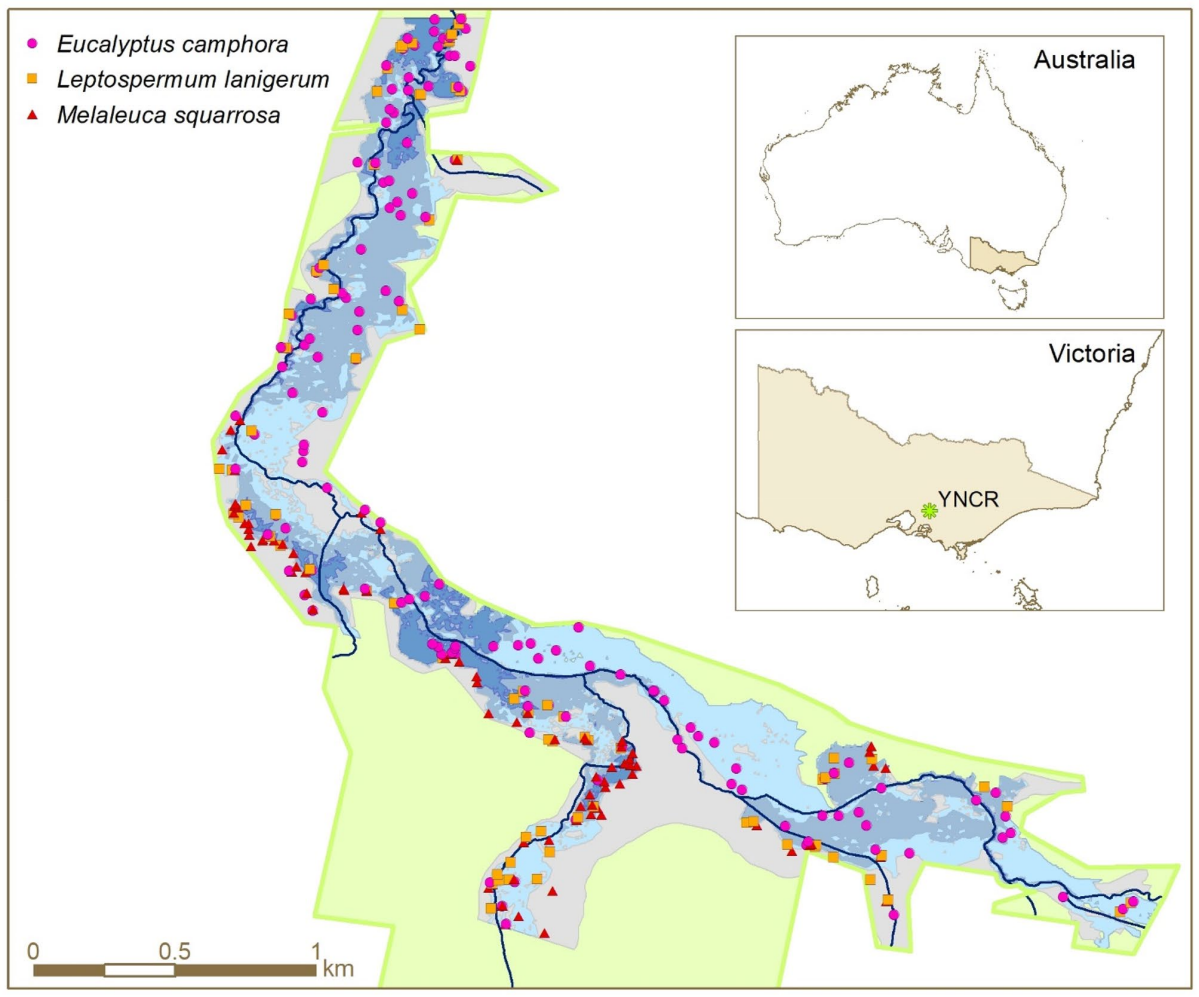
Map of all surveyed individuals of the three studied species within the Yellingbo Nature Conservation Reserve (green polygon). Shading represents flooding gradient categories used for sample point stratification (with grey indicating non-flood-prone areas and blues indicating flood-prone areas with darker blues representing higher flood-proneness). The map was generated in ArcMap version 10.5.1 (https://desktop.arcgis.com/).

Our study was undertaken in the Yellingbo Nature Conservation Reserve located around 45 km east of Melbourne, Victoria, Australia (Fig. 2). The reserve is embedded in an agricultural landscape and is around 640 ha comprising narrow riparian zones bordering local creeks. Low-lying floodplains along the Cockatoo and Macclesfield Creeks, which were focus of our surveys, are dominated by ‘Sedge-rich *Eucalyptus camphora* Swamp’ community^26^. These forests naturally experience seasonal to near-permanent inundation and vary in structure from open forest to woodland. The highly flood-tolerant mountain swamp gum *Eucalyptus camphora* is the sole overstorey species. The midstorey is dominated by thickets of woolly tea tree *Leptospermum lanigerum* and scented paperbark *Melaleuca squarrosa*, both of which are flood tolerant small trees or shrubs^27^. The largest remnants of this forest type are found within the Yellingbo Nature Conservation Reserve where they suffer dieback as a result of past human alterations of local watercourses^28^. The long-term survival of this threatened riparian forest likely depends on management interventions. Despite thorough documentation of declining tree and shrub condition, the ecology of the three major woody species is not well understood^29,30^.

### Survey design

We confined the survey area to elevations lower than 120 m above sea level as *Eucalyptus camphora* swamp does not occur above this elevation within the reserve^27^. Only areas mapped as vegetation communities containing the studied species were included. The survey area was further limited to match the extent of a hydrological model (see below) and was in total 1.69 km^2^.

In order to ensure that survey points were distributed across the hydrological gradient, we simulated different sized flooding events using a hydrologic model (described below). The spatial extents of these events were then used to classify the study area into four broad flooding categories. Flooding categories one, two and three comprised areas which were flooded by low, medium and high flow events, respectively. Flooding category one represents the wettest parts of the floodplain whereas categories two and three are less frequently flooded. Flooding category four contained the rarely inundated parts of the survey area that remained unflooded in the modelled flow events.

To equally represent all flooding categories, we used a stratified random sampling approach. We generated 40 random coordinates within the area represented by each category and four additional points per point were generated as spares in case some positions were unsuitable for sampling.

During field surveys, we visited locations by navigating with a handheld GPS device (Garmin etrx30) to the predefined points. From there, we surveyed the nearest individual of each of the species *E. camphora*, *M. squarrosa* and *L. lanigerum* and mapped their actual geographic position. If no tree was found within a radius of 10 m of a given sample point, we visited the closest point from the spare dataset instead. If no individual was present near any of the four closest spare points no tree was recorded at the location.

After visiting all of the original points (including the four extra points) additional points were generated randomly in the areas where the two shrub species were found during the course of previous sampling. We thereby increased sample size for each of these less widespread species to approximately 20 individuals per flooding category. We conducted all surveying and tree and shrub measurements from March to April 2018 to take advantage of low water levels and therefore best accessibility. In total, we sampled 292 trees comprising 133 *E. camphora*, 78 *L. lanigerum* and 84 M*. squarrosa*.

### Tree surveys

Elongated stems are a major feature of woody plants defining their overall architecture. To characterise and compare growth habits, we measured diameter at breast height (DBH) and height (to highest live foliage) of each tree and shrub. In some cases (for 21/133 *E. camphora* and 1/84 *L. lanigerum*) visibility impairment precluded height measurement via clinometer. For multi-stemmed individuals, we counted all stems, measured their DBH, and determined height of the tallest one. To yield crown width we measured maximum crown diameter and perpendicular crown diameter for every sampled plant and calculated the mean.

Flooding and associated unstable, boggy substrates might deter trees from the usual vertical growth and force them into leaning positions. Thus, we recorded inclination angle of the main stem (at DBH level) relative to vertical using a protractor.

The emergence of epicormic sprouts, be it a symptom of stress or sign of recovery^31^, is a common reaction to disturbance and reflects a tree’s ability to regenerate vegetatively. We estimated epicormic growth using a scale from 0 to 3 indicating absent, scarce, common or abundant expression of epicormic growth^32^.

Using the same scale, we assessed sexual reproduction by estimating the combined relative abundance of reproductive structures, namely buds, flowers and capsules. Flowers indicate only current reproductive activity and not all species were flowering during the fieldwork campaign. Owing to serotiny and the long timespan for bud crop development, different developmental stages of reproductive structures (current and past reproduction) can appear simultaneously on a single tree.

Growth and reproduction may both be affected by plant condition, for which crown vigour has been proven as a suitable and rapid measure^22,31,33^. For each sampled plant, we assessed crown vigour by visually estimating the proportion of the potential crown supporting live foliage to the nearest 5%.

Moreover, growth rates and tree shapes can be significantly influenced by competition. For each sampled tree or shrub, we therefore measured the distances to its nearest four neighbours, one in each compass quadrant and calculated the average nearest-neighbour distance. For *E. camphora*, only neighbouring trees were included, whereas for *L. leptospermum* and *M. squarrosa*, both trees and shrubs were considered neighbours.

### Hydrologic modelling

The surveyed floodplain area has a very low elevational gradient such that floods are low energy and geomorphology does not vary greatly across the system. As such, we did not explicitly examine geomorphology in this study and focused on hydrology. After completing tree surveys, we determined local flood regime history for each study tree using the output of a grid-based, 2-dimensional hydrological model built in TUFLOW classic (www.tuflow.com), which was calibrated with recent water-level data from four sites within the study area. The model generated historic-flow series (1998–present) of water levels across the study area with a 5-m grid-cell spatial resolution and a daily temporal resolution. See Greet et al.^34^ for more details.

We extracted water-level time series for each surveyed tree and shrub from the model output. Using the recorded coordinates, individual water-level data were extracted for the grid cell in which the respective tree or shrub was located. Some individuals that were located next to the stream were allocated to a grid-cell that the model designated as the stream channel, resulting in them being erroneously characterised as permanently inundated. In these cases, water level data was extracted for the eight surrounding cells. We then excluded those that were also permanently inundated, and the average of the remaining cells was used to create a water level time series for that individual.

To characterise the flood regime history for each tree, we considered water levels of zero as dry and values greater than zero as inundated. Therefore, the first day with a water level greater zero marked the start of a flooding event and the reduction to zero the end of the respective event. Consequently, the number of consecutive days of flooding defined the length of a flooding event.

**Figure 3:**
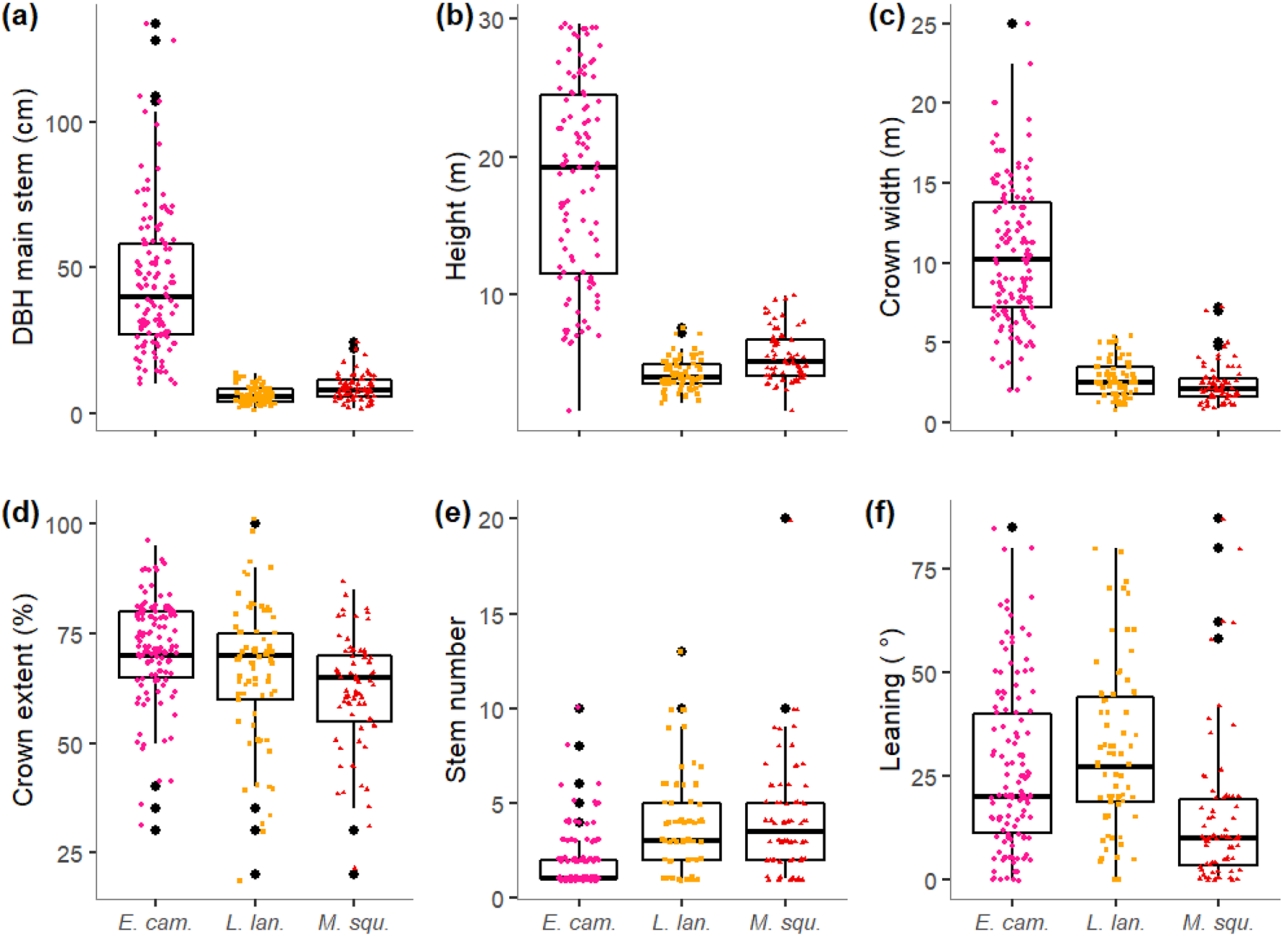
Boxplots of main stem DBH (**a**), plant height (**b**), crown width (**c**), crown extent (**d**), stem number (**e**) and stem leaning (**f**) for surveyed plants of the three studied species. Coloured points indicate raw data (n = 129 for *E. camphora*, n = 75 for *L. lanigerum*, n = 74 for *M. squarrosa* for all variables except for plant height for which n = 108 for *E. camphora* and n = 74 for *L. lanigerum*). *DBH* diameter at breast height, *E. cam* = *E. camphora*, *L. lan* *= L. lanigerum*, *M. squ* = *M. squarosa*. The figure was generated in R version 3.5.0 (https://www.r-project.org/).

We calculated the following flood regime metrics for the modelled 20-year period (1998–2018): mean and maximum length of flooding periods, mean and maximum length of dry spells (not inundated periods), the mean length of flooding periods during the growing season (November–June), the average number of flooding events per year and mean flooding depth. All variables were skewed and thus log-transformed before we tested for correlation (Online Resource 1, Fig. SM1). Although flood magnitude has been found to affect herbaceous riparian vegetation in other systems^35^, we assumed the observed flooding magnitude, i.e. mean water levels (mean = 0.06 cm, median = 0.01 cm, max = 0.92 cm), to be less important for the relatively tall trees and shrubs studied here (Fig. 3b). We further assumed maximum values to be less influential for tree and shrub growth over long periods. Hence, we selected two contrasting aspects characterizing long-term flood regime as predictors for further statistical analysis. These were the mean length of flooding events and the average number of flooding events per year representing duration and frequency of flooding. They were not strongly correlated with each other (Pearson correlation coefficient = 0.18). Both flooding duration and frequency have been shown to impact tree development in riparian ecosystems^36,37^.

14 out of 292 sampled trees from across the study area were excluded from statistical analyses due to model outputs suggesting unrealistic high flood duration (i.e. mean inundation duration > 500 days) or frequency (i.e. > 300 events), likely owing to errors of local topography representation based on our field observations.

### Statistical analysis

We performed multiple regression separately for each of the three species to:

1. Assess the strength of relationships between flood regime (flood frequency, flood duration) and tree and shrub morphology (DBH of main stem, height, crown width, stem number, leaning and crown extent); and flood regime and reproductive strategy (the extent of sexual reproduction and epicormic growth), thereby testing hypothesis 1 and 2 (H1 and H2); and
2. assess the relationships between morphology and both reproduction types, testing hypothesis 3 (H3).

For each analysis we used hierarchical partitioning to identify those variables which independently explained the most variance in morphology and reproduction, respectively.

First, we tested how much variation in morphology and reproductive strategy variables was explained by each of the two flood regime variables (H1 and H2). We fit 8 generalised linear models (response variables: main stream DBH, height and crown width, stem number, leaning, crown extent, sexual reproduction, and epicormic growth; predictor variables: flood frequency, flood duration). We chose the appropriate distribution used in the linear model for each variable (Table 1). Beta regression was undertaken using the *betareg* package^38^ and ordinal regression using the *MASS* package^39^.

**Table 1:** Measured morphology and reproduction variables and distribution for model fitting.

Tree measurements	Unit	Distribution
**Morphology variables**		
Main stem DBH	cm	Gaussian
Height	m	Gaussian
Crown with	m	Gaussian
Crown extent	%	Beta
Number of stems	Integer	Poisson
Leaning	°	Beta
**Reproductive strategy variables**		
Sexual reproduction	Score 0–3	Binominal
Epicormic growth	Score 0–3	Binominal

We initially included the average nearest neighbour distance (a surrogate for competition) in models predicting morphology variables (H1). However, we later omitted this additional predictor as it generally did not increase the proportion of explained variance (Online Resource 2, Table SM1).

To assess how much variation in reproductive strategy variables was explained by morphology variables (H3), for each species, we calculated two additional linear models for the response variables of sexual reproduction and epicormic growth with each six predictor variables (main stem DBH, height, crown width, stem number, leaning and crown extent). Both of these models used a binomial distribution adapted for ordered factors.

For each model, we used hierarchical partitioning of log-likelihood values using the hier.part package^40^ to determine the proportion of explained variance explained independently by each predictor variable^41^. This method allows identification of variables that have a strong independent correlation with the dependent variable, in contrast to variables that have little independent effect but have a high correlation with the dependent variable resulting from joint correlation with other predictor variables. Variables that independently explained a larger proportion of variance than could be explained by chance were identified by comparison of the observed value of independent contribution to explained variance (I) to a population of Is from 1000 randomizations of the data matrix. Significance was accepted at the upper 95% confidence limit (Z score > 1.65: Mac Nally^42^, Mac Nally and Walsh^40^).

To assess the goodness of fit for each model, we present R^2^ or pseudo-R^2^ values (according to Nagelkerke using the DescTools package: Signorell et al.^43^) for ordinal regression and Ferrari and Cribari-Neto^62^ for beta regression, respectively. We considered variables with a total contribution to explained variance (i.e. proportion explained × R^2^) > 0.05 to be influential variables and the direction of their effect important.

Lastly, we performed a PCA analyses and ordination to assess associations between different morphology attributes and reproduction variables across all species (H3).

All statistical analysis was performed in R version 3.5.0^44^.

## Results

All species were similarly distributed across the hydrological gradient based on flood duration and frequency (Online Resource 3, Fig. SM2, Online Resource 4, Table SM2). However, *E. camphora* was more spatially widespread, whereas both shrub species had patchier distributions within the study area (Fig. 2). Three morphology attributes (main stem DBH, tree height, and crown width) were more variable for the taller species *E. camphora* than for *L. lanigerum* and *M. squarrosa* (Fig. 3a–c, Online Resource 4, Table SM2). Condition varied widely in all species (Fig. 3d). Multi-stemmed growth was more common in the shrub species than *E. camphora* (Fig. 3e). Stem leaning was generally frequent albeit to varying degrees (Fig. 3f). More than 90% of trees and shrubs showed some evidence of sexual reproduction (Online Resource 5, Fig. SM3a). Such evidence was common or abundant in 60% of *E. camphora* trees surveyed, but in both shrub species, evidence of sexual reproduction was mostly scarce (51% in *L. lanigerum*, 73% in *M. squarrosa*, Online Resource 5, Fig. SM3a). Epicormic growth was prevalent in all species with more than 50% of individuals of all three species displaying common or abundant epicormic growth (Online Resource 5, Fig. SM3b). *M. squarrosa* exhibited the highest extent of epicormic growth (68% common or abundant).

### H1 and H2: Association between flood regime and plant morphology and reproduction

Flood regime explained a modest amount of variation in morphology and reproductive strategy variables (maximum R^2^ of 0.27 for *M. squarrosa* stem number, Table 2), providing partial support for H1 and H2. More associations between flood regime and morphology and reproduction were found for *E. camphora* (six variables) than for *L. leptospermum* and *M. squarrosa* (two and three variables, respectively).

**Table 2:** Explained variance of models of morphology and reproductive strategy variables as a function of flood frequency and duration.

	*E. camphora*		*L. lanigerum*		*M. squarrosa*	
	dir	var	dir	var	dir	var
**Morphology variables**						
Main stem DBH						
R^2^		**0.07**				
Frequency	−	0.75*				
Duration		0.25				
Height						
R^2^		**0.13**				**0.05**
Frequency	−	0.90*				0.17
Duration		0.10				0.83
Crown width						
R^2^				**0.05**		**0.05**
Frequency				0.80		0.83
Duration				0.20		0.17
Crown extent						
Pseudo-R^2^						
Frequency						
Duration						
Number of stems						
R^2^		**0.10**		**0.10**		**0.27**
Frequency	+	0.90*		0.03	+	0.47*
Duration		0.10	+	0.97*	+	0.53*
Leaning						
Pseudo-R^2^		**0.11**		**0.13**		**0.20**
Frequency	+	0.57*	+	0.81*		0.20
Duration	+	0.43*		0.19	+	0.80*
**Reproductive strategy variables**						
Sexual reproduction						
Pseudo-R^2^		**0.13**				
Frequency	−	0.90*				
Duration		0.10				
Epicormic growth						
Pseudo-R^2^		**0.07**				**0.07**
Frequency	+	0.83*				0.04
Duration		0.17			+	0.96*

Explained variance (in bold) is expressed as R^2^ or pseudo-R^2^ as appropriate. For each predictor variable, its proportional independent contribution is shown, and, if its total contribution to explained variance (i.e. proportion explained × R^2^) > 0.05, the direction of the effect (dir) is indicated as positive, “+”, or negative, “−”. Variables that independently explained a larger proportion of variance than could be explained by chance are marked with an asterisk. Sample sizes were n = 129 for *E. camphora*, n = 75 for* L. lanigerum*, n = 74 for M. squarrosa for all response variables except for plant height for which n = 108 for *E. camphora* and n = 74 for *L. lanigerum*. *DBH* diameter at breast height, *dir* direction of effect, *var* proportion of explained variance (bold) and contribution to explained variance, respectively.

**Figure 4:**
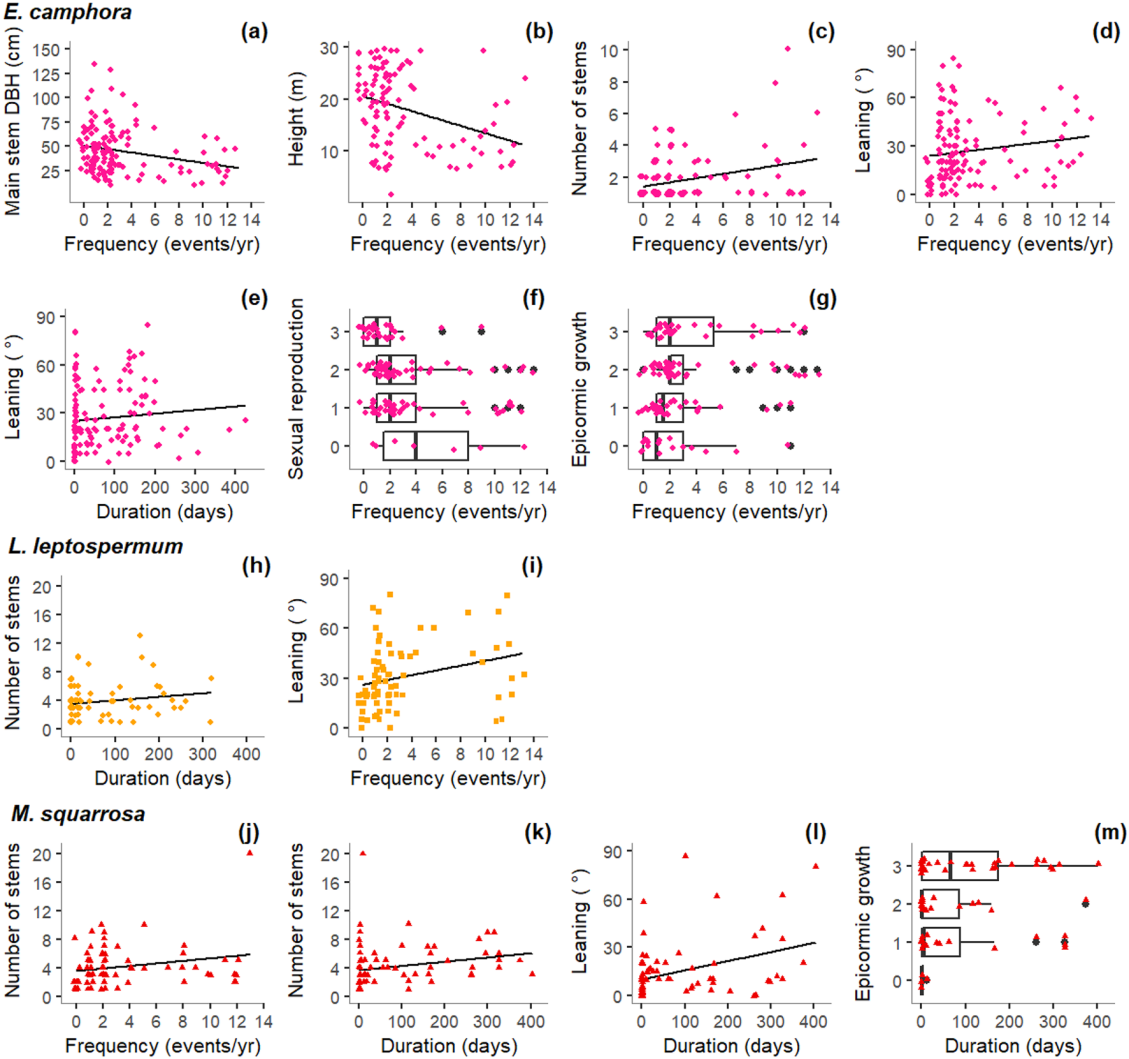
Relationships of flood duration and frequency and measured morphology variables, sexual reproduction and epicormic growth for *E. camphora*, *L. lanigerum* and *M. squarrosa*. Only relationships from models with R^2^ or pseudo R^2^ > 0.05 and for predictor variables with a significant independent contribution to the overall explained variance > 0.30 are shown. Note that plots show untransformed values for flood duration and frequency whereas statistical modelling was undertaken using log transformed values. Points indicate raw data (n = 129 for *E. camphora*, n = 75 for *L. lanigerum*, n = 74 for *M. squarrosa*). Frequency (events/year) = mean number of flooding events per year; duration (days) = mean flooding event duration; sexual reproduction = score for sexual reproduction; epicormic growth = score for epicormic growth. The figure was generated in R version 3.5.0 (https://www.r-project.org/).

Flood frequency was a consistently strong contributor to explained variance in growth-form variables and reproduction in *E. camphora* (Table 2). Trees experiencing more frequent flooding tended to have thinner main stems (Fig. 4a), be shorter (Fig. 4b) and have greater numbers of stems (Fig. 4c), and they tended to lean more (Fig. 4d), less commonly show evidence of sexual reproduction (Fig. 4f) and display more epicormic growth (Fig. 4g). Leaning was the only variable for which flood duration had a strong independent contribution to explained variance. Stem leaning was more pronounced in *E. camphora* in locations with longer periods of flooding (Table 2, Fig. 4e).

For the shrub species, flood duration was more commonly a strong contributor to explained variance. Both *L. lanigerum* and *M. squarrosa* subjected to longer flooding tended to have more stems (Fig. 4h,k), and *M. squarrosa* tended to lean more and have more epicormic growth in locations with longer floods (Fig. 4l,m). In addition, flood frequency was a strong contributor to explained variance for leaning of *L. lanigerum* (more leaning with more frequent floods, Fig. 4i), and for stem number of *M. squarrosa* (more stems with more frequent floods, Fig. 4j).

We did not find evidence for an effect of flood duration or frequency on the condition (crown extent) or crown width of any species (Table 2).

### H3: Associations between morphology and reproduction type and extent

For all species, a larger proportion of variation in reproduction was explained by morphology than by flood regime (Tables 2, 3). In general, main stem DBH, stem leaning, and crown extent had high contributions to the explained variance in sexual reproduction and epicormic growth, providing support for H3 (Table 3).

**Table 3:** Explained variance of models of reproductive strategy variables as a function of growth-form variables.

	*E. camphora*		*L. leptospermum*		*M. squarrosa*	
	dir	var	dir	var	dir	Var
**Sexual reproduction**						
Pseudo R^2^		**0.18**		**0.16**		**0.28**
Main stem DBH	+	0.64*	+	0.36*		0.13
Height	+	0.57	−	0.53	−	0.23*
Crown width		0.13		0.10	+	0.92
Stem number	−	0.70	−	0.41		0.10
Leaning	+	0.26	−	0.32*	−	0.82
Crown extent	+	0.79		0.12	+	0.36*
**Epicormic growth**						
Pseudo R^2^		**0.25**		**0.13**		**0.16**
Main stem	−	0.63		0.21	+	0.46*
Height	−	0.88		0.12		0.22
Crown width		0.14		0.11	+	0.29*
Stem number	+	0.56	+	0.70		0.17
Leaning	+	0.76*		0.31		0.19
Crown extent	−	0.21	−	0.47*	−	0.33

Explained variance (in bold) is expressed as pseudo-R^2^. For each predictor variable, its proportional independent contribution is shown, and, if its total contribution to explained variance (i.e. proportion explained × R^2^) > 0.05, the direction of the effect (dir) is indicated as positive, “+”, or negative, “−”. Variables that independently explained a larger proportion of variance than could be explained by chance are marked with an asterisk. Sample sizes were n = 111 for *E. camphora*, n = 76 for *L. lanigerum* and n = 77 for *M. squarrosa*. *DBH* diameter at breast height. *Var* proportion of explained variance (bold) and contribution to explained variance, respectively.

**Figure 5:**
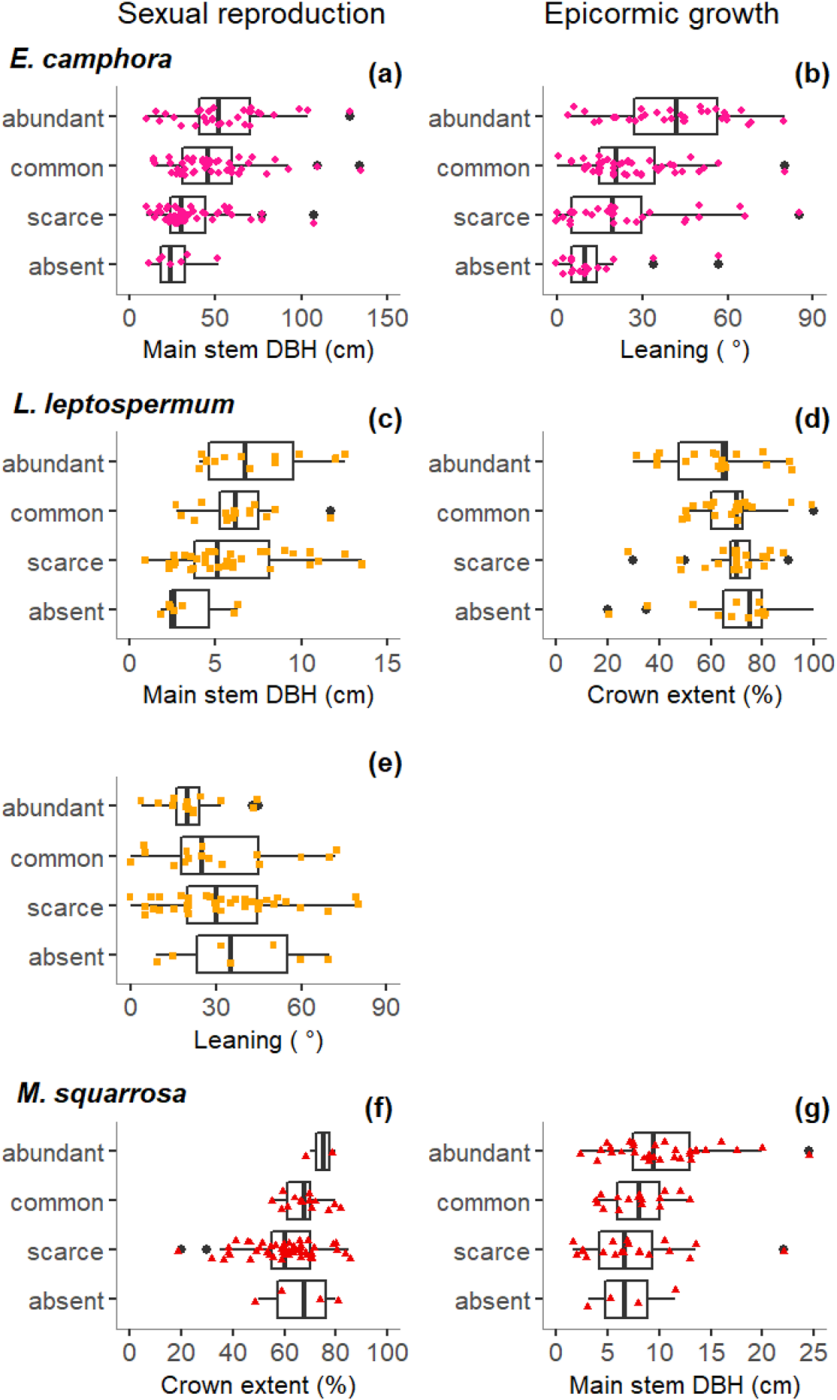
Relationships between morphology variables and sexual reproduction and epicormic growth for *E. camphora*, *L. leptospermum* and *M. squarrosa*. Only relationships for predictor variables with a significant independent contribution > 0.30 to the explained variance are shown. Points indicate raw data (n = 111 for *E. camphora*, n = 76 for *L. lanigerum*, n = 77 for *M. squarrosa*). DBH = Diameter at breast height. The figure was generated in R version 3.5.0 (https://www.r-project.org/).

Individuals with higher main stem DBH showed higher extents of sexual reproduction in *E. camphora* and *L. leptospermum* (Fig. 5a,c, Table 3), whilst *M. squarrosa* with larger stem diameters had more epicormic growth (Fig. 5g, Table 3).

Higher degrees of stem leaning were associated with less sexual reproduction in *L. leptospermum* (Fig. 5e, Table 3) and more epicormic growth in *E. camphora* (Fig. 5b, Table 3).

*M. squarrosa* in better condition had higher extents of sexual reproduction (Fig. 5f, Table 3), whereas *L. leptospermums* in lower condition had more epicormic growth (Fig. 5d, Table 3).

The first two axes of the PCA explained 56.1% of the variation in morphology and reproduction attributes of all three species and show the attributes of the larger tree species, *E. camphora,* to be somewhat distinct from the other two species (Online Resource 6, Fig. SM4). Across the three species, the two reproductive strategies are negatively correlated and associated with divergent morphology which provides further support for H3. Multi-stemmed form and stem leaning is associated with asexual reproduction (epicormic growth), while sexual reproduction is associated with greater plant size (main stem DBH and height).

## Discussion

By examining relationships between flood regime, morphology and reproductive strategy of three dominant riparian woody plants, we found support for all of our three hypothesis: flood frequency and duration influenced woody riparian plant morphology (H1) and reproduction (H2), and different morphology was associated with different reproductive strategies (H3). Increased flooding generally resulted in increased stem numbers and greater stem leaning of our study species; this morphology was associated with asexual reproduction. More frequent flooding also reduced size and sexual reproduction in *E. camphora*. Sexual reproduction was more common in taller plants with single, more upright stems in good condition. Compared with research that examines plant responses to other disturbances our findings suggest that woody plants respond to physical disturbance in a similar set of ways regardless of the nature of that disturbance—be it fires, hurricanes, avalanches or moderate, regular disturbance by floods^18,19,21^. Interestingly, the morphology and reproduction of our three study species were affected by different aspects of the flood regime (flood frequency cf. duration). This indicates that flood regime (i.e. variable frequency and duration of flooding events) is critical to the structural integrity and self-maintenance of species-diverse riparian forests.

### H1: Morphology is plastic in response to flooding

Apart from being a stress response and survival mechanism, the morphological plasticity we found in our study species may constitute an adaptation to mild or chronic disturbance. Evergreen species that do not have marked seasonal growth interruptions may lack the ability to avoid flood stress via dormancy and thus require a high level of adaptation to cope with physical and physiological stress^45^. Adaptive traits conferring flood tolerance and the physiological mechanisms underpinning morphological changes are manifold^8^, and co-occurring species may respond to different attributes of the flood regime^46^. In our study the more flood tolerant species, *L. lanigerum* and *M. squarrosa*^30^, seemed less sensitive to frequent flooding and their morphology responses tended to occur with longer duration floods. Conversely, *E. camphora* initiates more immediate responses to flooding^29^ and its morphological variability was more strongly associated with flood frequency. As documented elsewhere, we found the morphology of shrubs to be less impacted by disturbance than trees^21^, but also to be less variable than trees overall. Growth rates of the shrub species studied here (*L. lanigerum* and *M. squarrosa*) were previously shown to respond in a non-linear manner to increased water levels^30^ and this may also apply to their morphology responses.

Consistent with patterns found in other wetlands, we found an increased prevalence of multi-stemmed growth with increased flooding^4,36,47^. Although many plants possess the ability to resprout, resprouting typically requires environmental disturbance^24^. This often follows, but does not necessarily require, visible plant damage^48^. Suppressed root and shoot growth during flooding or mechanical stress may alter internal hormone balance^14,48^. Consequently, reduced apical dominance, the hormonally regulated prevailing growth of the main stem, facilitates emergence of epicormic sprouts^49^ and therefore stem multiplication. A larger number of stems increases an individual’s surface area usable for stem gas exchange and therefore potentially enhances oxygen transport to roots during soil anoxia under inundation^4^. Besides, possessing a plurality of usually smaller stems reduces the mortality risk due to stem damage, in part because smaller stems are less prone to mechanical perturbation^50^.

Repeated physical perturbation from flowing water and prolonged waterlogging likely cause stem leaning of woody plants, such as we observed. As shown in other disturbance prone ecosystems, the upright growth of woody stems can be compromised by external mechanical stresses such as avalanches or substrate movements^20,21^. In addition, longer flood durations induce soil softening and thus can diminish anchorage and, while preventing excessive root breakage, cause plant tilting. This might especially be the case in combination with strong winds. Thereby larger species, such as *E. camphora* (but also *M. squarrosa)*, provide more surface area and are likely exposed to higher wind speeds and are therefore more vulnerable to being blown over.

Slow growth, compromised stability and altered morphology due to flooding might limit the size of *E. camphora*, the tallest of our study species. A typical characteristic of the tree growth habit is the pursuit of reaching large size^1^. However, tree growth is typically hampered by prolonged flooding^34^, as has been previously observed in *E. camphora* when subjected to inundation in the field^51^ and in greenhouse experiments^29^. Accordingly, we found that the size of *E. camphora* was inversely related to flood frequency, with smaller plants growing in areas with higher flood frequencies. Particularly under reoccurring flooding disturbance, increase in size requires a larger proportion of photosynthetic assimilates to be invested in structural support tissue^52,53^, which in turn slows growth^50^. Whereas shrubs grow multi-stemmed even in the absence of disturbance, the distribution of biomass across multiple stems in frequently flooded trees requires resource allocation changes at the cost of height growth^54^.

### H2: Flooding induces reproductive strategy responses

The extent of sexual reproduction decreased in *E. camphora* with increased flood frequency. Perhaps because high maintenance costs, especially of larger species during or post disturbance, have diverted resources away from the development of reproductive structures^55^. Diminished sexual reproduction might have indirectly resulted from retarded growth due to flooding. The period of growing tall is a phase of low reproduction in trees^53^. The reproductive phase is usually only entered after reaching a certain size^1^. *E. camphora* at regularly flooded sites probably mature slowly. Shrub species in contrast mature at smaller heights^12^. The size of *L. leptospermum* and *M. squarrosa* was not impacted by flooding and likewise, neither was their sexual reproduction; yet, sexual reproduction in these shrubs was generally low.

We found evidence that flooding encourages epicormic growth in *E. camphora* and *M. squarrosa*, which might compensate for absent or limited sexual reproduction. Such vegetative reproduction, potentially leading to multi-stemmed growth forms, is likely an essential survival mechanism, enabling rejuvenation, longevity and persistence where recruitment success is low^24^.

Our survey of both reproduction types reflected recently developed structures and this may explain the relatively weak correlations with long-term flooding patterns we found. Unlike in fire prone ecosystems, disturbances in riparian forests are typically milder, plant damage less severe and post disturbance conditions less homogenous. Consequently reproduction responses in riparian trees and shrubs may be more diverse and less obvious^9^.

### H3: Reproductive strategy is associated with morphology

Additional to environmental drivers, reproduction is regulated by intrinsic biological factors and hence we found plants with contrasting morphology displayed contrasting reproductive strategies. Asexual and sexual reproduction are thought to be traded-off against each other as resources required for resprouting or resources stored to maintain resprouting ability are diverted from the production of reproductive structures and vice versa^9,56^. Consistent with observations from many woody species, we found sexual reproduction increased with stem size in *E. camphora* and *L. leptospermum*^57,58^. Investment in reproduction might increase only after decelerating size increase^1^ and larger individuals are more able to expose seeds to dispersal vectors^59^. Plants struggling with stress may avert resources from flower, fruit and seed production^22^. Accordingly, we found diminished sexual reproduction in leaning *L. leptospermum* and in *M. squarrosa* in poorer conditions indicated by their crown extent.

Stem leaning is likely ecologically significant for the persistence and reproduction of *E. camphora* with its treefall often resulting in new individuals^51^. The diversion from vertical to diagonal or horizontal orientation can initiate trunk suckering and trigger the emergence of epicormic shoots^20^. Epicormic growth serves to reconstruct lost biomass and optimize leaf positioning and light harvest post disturbance. It can also be triggered by hormonal changes following partial dieback^22^ and accordingly we found more epicormic growth in *L. leptospermum* with declining condition. A requirement for epicormic growth is the prevalence of stored resources as well as buds to resprout from^56^. For both, bigger stems can provide a larger repository which might be the reason *M. squarrosa* with bigger stems displayed more epicormic growth.

Unsurprisingly, and similar to many ecological field studies^10,60^, a large proportion of the variation in morphology and reproduction remained unexplained by the assessed flood regime variables and morphological attributes. Plant life processes, including morphology adaptations and reproduction, respond to and are determined by a multiplicity of interacting biotic and abiotic factors, which likely contributed noise to our data. Albeit simplifying complex water flows and fluxes, the modelled long-term hydrological data at the individual tree level enabled high accuracy in characterizing flood regime which is novel within ecological surveys^34^. This allowed us to uncover the diversity of functional responses of co-occurring species to flooding, despite the complexity of the riparian environment.

## Conclusion

Understanding the way in which hydrology impacts tree morphology and reproduction is crucial to predict vegetation changes in a world with ever-increasing waterway modification. Among the multiple drivers of degradation of riparian ecosystems, hydrology is one with the potential to be remediated by hydro-geomorphic restoration (e.g. dam modification or controlled flooding). The structural complexity and integrity of temperate riparian forests does not solely emerge from the presence or absence of its main species but also requires appropriate flood regimes. Morphology supported by flooding, e.g. multi-stemming and stem leaning, create dense stands and therefore specific microclimates and habitats for swamp adapted flora and fauna^51,61^. Our findings highlight that natural flooding regimes (i.e. frequent and longer duration floods) are also pivotal for the initiation and long-term success of vegetative reproduction, an important component of the self-maintenance of riparian woody species.

## Supplementary Information

Supplementary Information 1.

## Data Availability

The data that support the findings of this study are available from the corresponding author upon reasonable request.

## Code availability

All code for analysing the data associated with the submission/ article is available from the corresponding author upon reasonable request.
