# Supplementary material for: Flood disturbance affects morphology and reproduction of woody riparian plants
Source: Sci Rep. 2021 Aug 13;11:16477. doi: 10.1038/s41598-021-95543-0 (PMC8363665; doi:10.1038/s41598-021-95543-0)
Supplement: Supplementary file 1 — Supplementary Information 1. [file 41598_2021_95543_MOESM1_ESM.pdf]

Online Resource 1

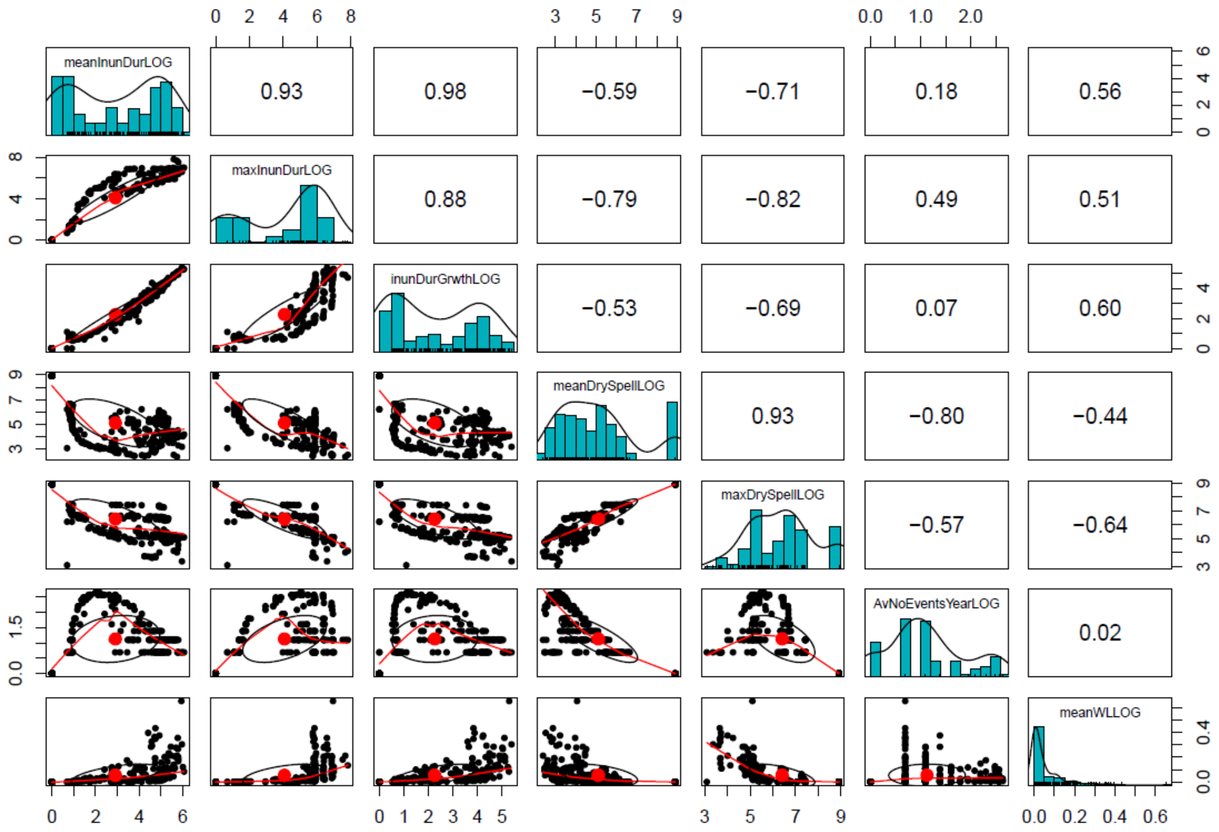

Fig. SM1 *Correlation matrix (Pearson's correlation coefficient) between mean flood duration (meanInunDurLOG), maximum flood duration (maxInunDurLOG), flooding during growth period (inunDurGrwthLOG), mean length dry periods (meanDrySpellLOG), maximum length dry periods (maxDrySpellLOG), average number of flooding events per year (AvNoEventsYearLOG) and mean water depth (meanWLLOG) of a 20-year period for all trees and shrubs (not distinguishing between species). All variables are log transformed. The figure was generated in R version 3.5.0 (https://www.r-project.org/).*

FLOOD DISTURBANCE AFFECTS MORPHOLOGY AND REPRODUCTION OF WOODY RIPARIAN PLANTS

Authors: Sarah Fischer, Joe Greet, Christopher J. Walsh, Jane A. Catford

Contact details for corresponding author: Sarah Fischer, rettichretter@hotmail.de, 0061448049172

Online Resource 2

Table SM1 Explained variance of models of growth-form and reproductive strategy variables as a function of flood frequency and duration. Explained variance (in bold) is expressed as R^2 or pseudo-R^2 as appropriate, and results are shown only for those models which explained ≥0.07 of the variance. For each predictor variable, its proportional independent contribution is shown, and, if its total contribution to explained variance (i.e. proportion explained x R2) > 0.05, the direction of the effect (dir) is indicated as positive, "+", or negative,"–". Variables that independently explained a larger proportion of variance than could be explained by chance are marked with an asterisk. Sample sizes were n = 129 for E. camphora, n = 75 for L. lanigerum, n = 74 for M. squarrosa for all response variables except for plant height for which n = 108 for E. camphora and n = 74 for L. lanigerum. DBH = Diameter at breast height. dir = direction of effect. var = proportion of explained variance (bold) and contribution to explained variance, respectively.

	E. camphora		*L. lanigerum*		*M. squarrosa*	
	dir	var	dir	var	dir	var
Height						
Full model R^2		**0.13**		**0.05**		**0.08**
Frequency	-	0.90*		0.34		0.11
Duration		0.10		0.46		0.56
Competition		0.00		0.20		0.33
DBH main stem						
Full model R^2		**0.10**				
Frequency	-	0.51*				
Duration		0.19				
Competition		0.29*				
Crown width						
Full model R^2				**0.13**		**0.05**
Frequency				0.26		0.83
Duration				0.05		0.17
Competition			+	0.69*		0.01

FLOOD DISTURBANCE AFFECTS MORPHOLOGY AND REPRODUCTION OF WOODY RIPARIAN PLANTS

Authors: Sarah Fischer, Joe Greet, Christopher J. Walsh, Jane A. Catford

Contact details for corresponding author: Sarah Fischer, rettichretter@hotmail.de, 0061448049172

Online Resource 3

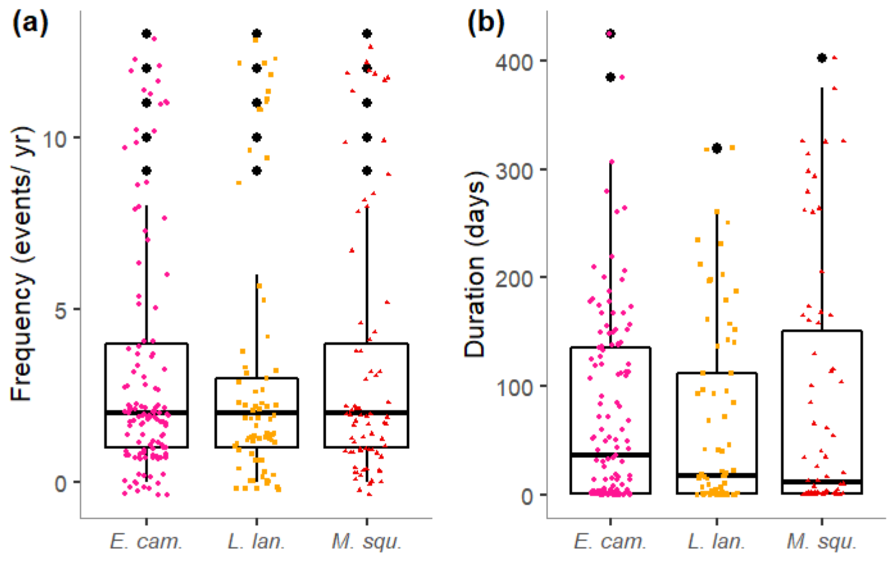

Fig. SM2 *Boxplots of flood frequency (a) and flood duration (b) for a 20-year timespan prior to our surveys for surveyed plants of the three studied species. Points indicate raw data (n = 129 for E. camphora, n = 75 for L. lanigerum,* n = 74 for *M. squarrosa*). *Frequency (events/*yr*) = mean number of flooding events per year, Duration (days) = mean flooding event duration. The figure was generated in R version 3.5.0 (https://www.r-project.org/).*

FLOOD DISTURBANCE AFFECTS MORPHOLOGY AND REPRODUCTION OF WOODY RIPARIAN PLANTS

Authors: Sarah Fischer, Joe Greet, Christopher J. Walsh, Jane A. Catford

Contact details for corresponding author: Sarah Fischer, rettichretter@hotmail.de, 0061448049172

Online Resource 4

Table SM2 Summary of morphology, competition, flood frequency, flood duration and mean water level for E. camphora, L. leptospermum and M. squarrosa.

	E. camphora			*L. lanigerum*			*M. squarrosa*		
	mean	SD	range	mean	SD	range	mean	SD	range
Height	18.20	7.34	1.6-29.63	4.24	1.14	2.10-7.56	5.85	2.32	1.6-14.77
DBH	44.89	24.43	10-134	6.19	3.08	0.90-13.50	8.77	4.51	1.6-24.5
Crown width	10.54	4.42	2.00-25.00	2.71	1.11	0.75-5.40	2.35	1.24	0.85-7.25
Stem number	1.84	1.49	1-10	3.84	2.59	1-13	4.12	3.02	1-20
Proportion dead stems	6.94	16.88	0-83.33	19.69	24.31	0-75.00	19.95	21.66	0-66.67
Leaning	26.76	19.96	0-85	30.59	19.67	0-80	14.78	17.97	0-87
Crown extent	71.94	11.53	30-95	66.27	15.71	20-100	61.89	12.81	20-85
Competition	11.25	11.69	1.18-74.18	4.01	2.74	0.60-16.00	2.88	2.72	0.53-21.63
Frequency	3.13	3/38	0-13	3.13	3.87	0-13	3.43	3.89	0-13
Duration	73.76	87.04	0-424.8	65.95	87.69	0-320.00	82.90	115.74	0-402.75
Mean Water level	0.05	0.10	0.00-0.54	0.07	0.11	0.00-0.45	0.06	0.12	0.00-0.92

FLOOD DISTURBANCE AFFECTS MORPHOLOGY AND REPRODUCTION OF WOODY RIPARIAN PLANTS

Authors: Sarah Fischer, Joe Greet, Christopher J. Walsh, Jane A. Catford

Contact details for corresponding author: Sarah Fischer, rettichretter@hotmail.de, 0061448049172

Online Resource 5

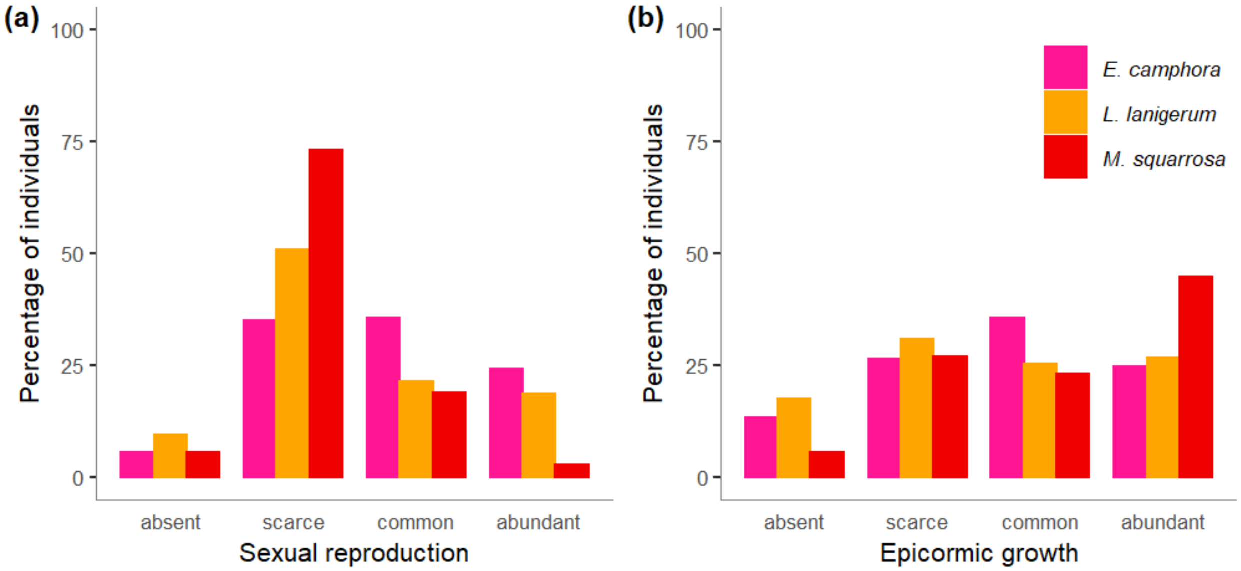

Fig. SM3 *Scores for sexual reproduction (a) and epicormic growth (b) for each studied species expressed as percentage of total individuals in each score category. n = 129 for E. camphora, n = 75 for L. lanigerum, n = 74 for M. squarrosa. The figure was generated in R version 3.5.0 (https://www.r-project.org/).*

FLOOD DISTURBANCE AFFECTS MORPHOLOGY AND REPRODUCTION OF WOODY RIPARIAN PLANTS

Authors: Sarah Fischer, Joe Greet, Christopher J. Walsh, Jane A. Catford

Contact details for corresponding author: Sarah Fischer, rettichretter@hotmail.de, 0061448049172

Online Resource 6

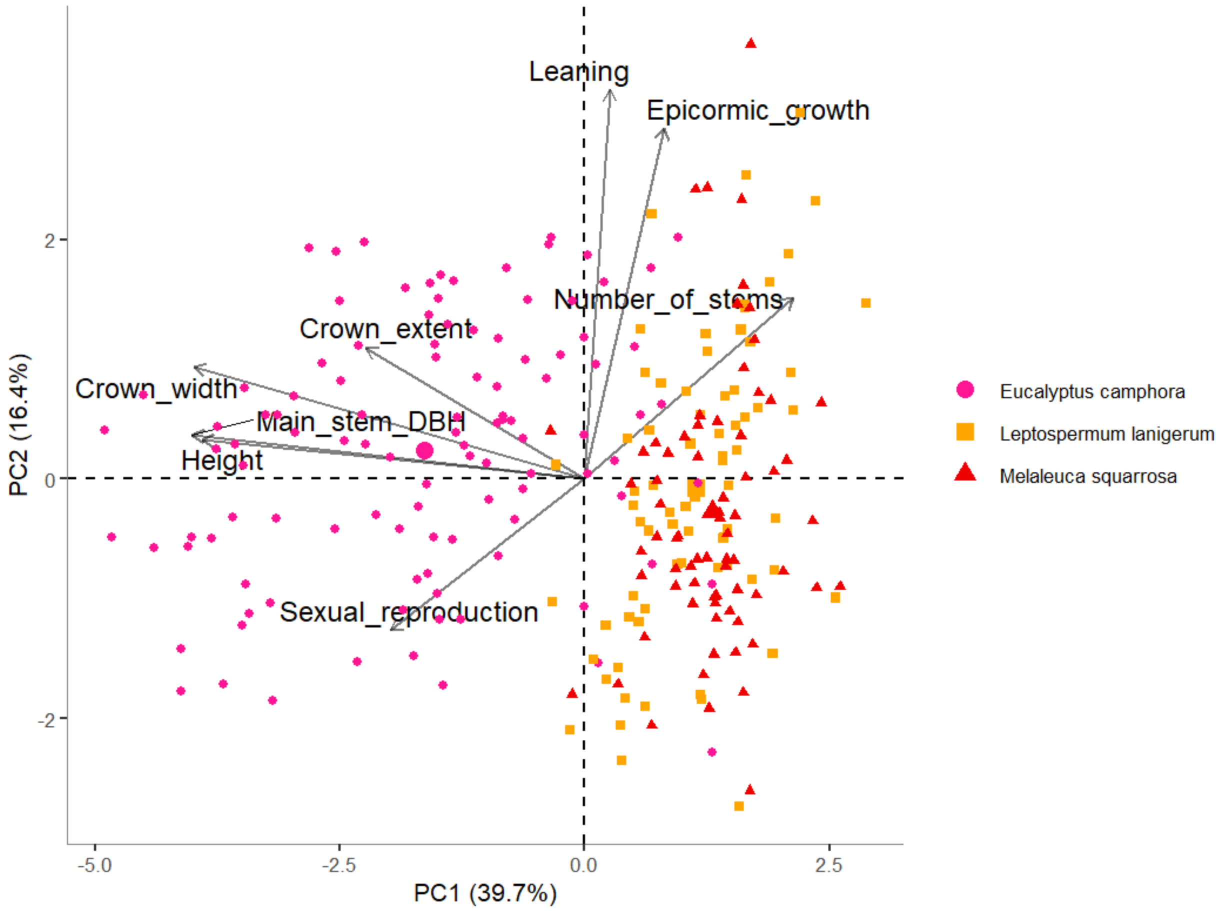

Fig. SM4 *PCA ordination of the six morphology and two reproductive strategy attributes across all three species. The figure was generated in R version 3.5.0 (https://www.r-project.org/).*

FLOOD DISTURBANCE AFFECTS MORPHOLOGY AND REPRODUCTION OF WOODY RIPARIAN PLANTS

Authors: Sarah Fischer, Joe Greet, Christopher J. Walsh, Jane A. Catford

Contact details for corresponding author: Sarah Fischer, rettichretter@hotmail.de, 0061448049172
